# The weight of the elephant(s): rethinking the tyranny of tradition in modern paramedic education

**DOI:** 10.29045/14784726.2026.9.11.2.2

**Published:** 2026-09-01

**Authors:** James Brogan

**Affiliations:** Robert Gordon University ORCID iD: https://orcid.org/0000-0002-1432-5661

September has arrived; a new ‘learning year’ dawns. For the most part, in the Western world, it is a return to studies, or the start of new learning journeys across primary, secondary, further and higher education (although it should be noted that there are always exceptions to the rules, such as primary and secondary schools returning mid-August in some parts of Scotland). At this point, it’s timely to consider ‘education’ within the paramedic profession and how education, professionalisation, framing and other factors, such as lived experience, location, local norms, customs and cultures, will shape future education. We need to take a long, hard look and ask ourselves some fundamental questions – we should start this by confronting the elephant(s) in the room.

## History of paramedic ‘training’

Historically, paramedic ‘training’ followed a well-worn path of traditional ambulance training, with a focus on functional, didactic, discrete skills, initially in the 1960s through the ‘Millar’ system (Millar, 1966, 1967) and later in the 1980s and 1990s through the Institute of Health Care Development (IHCD) publications (Brooks et al., 2016), which covered clinical, educational and driving curricula. For the focus of clinical ‘training’ for paramedics, we must recognise and consider the relevant manuals in this regard – the trinity of manuals that outline clinical and educational approaches to paramedic training. These are the Basic Training Manual (Green), the Paramedic Manual (Blue) – established in 1991 (Brooks et al., 2016) – and the Tutor Manual (Gold). In considering education, the ‘Gold book’ was the highway code for instructing and assessing all levels of ambulance worker, and it focused primarily and ruthlessly on delivery of training in ‘cognitive’ and ‘psycho-motor’ lessons, based and assessed against behavioural objectives, each with a required ‘performance’ (what the student should be able to do), a ‘condition’ (what they could / could not use, such as equipment, reference material etc.) and a ‘standard’ (often the relevant manual, dependent on the session, learners and environment). For example, ‘That the student should be able to demonstrate mechanical ventilation using a bag-valve-mask, as per the basic training manual’. As an interesting side note, only one half day out of many months of tutor training was spent looking at something known as ‘affective techniques’, that is, values, emotions and feelings.

## A tyranny of historical practice?

You might wonder why I have spent time outlining this approach; however, it is important to appreciate that this method of teaching (and indeed teaching future paramedic and ambulance tutors how to instruct) has been prevalent in ambulance and, more recently, paramedic training (and education) for decades. Indeed, even with the near-complete shift of pre-registration paramedic education from ambulance service internal training departments to higher education establishments in the UK, there are still elements of paramedic education today that continue to fetishise this functional, skills-based, didactic method of education, whether this is now being contained within pockets of higher education establishments, or indeed in the workplace post-registration through CPD, mandatory updates and occupation-specific training courses. Why?

There may be some justification for this style of ‘training’ – in places – but the question is where and at what level – what is the right ‘mix’? As noted in the discourse within, and subsequently flourishing from, the scholarly perspective forwarded by Corman et al. (2025), there is a need to have a wide-ranging, honest and deeply reflective conversation as to where training and education of paramedics has come from, where it is currently and where it needs to move in the future to meet the requirements of the modern demands upon the profession. Corman et al. (2025) discuss their contention of the ‘tyranny of the bio-psycho-medico’ and the ‘hidden and translucent curriculum’. While I do not intend to develop these arguments much further in this piece, I would commend all readers to examine the scholarly discourse emanating from this original piece and consider a few practical points as concepts to frame reflections upon.

There is rightly an expectation on those who interface in education-focused roles, such as academics teaching on higher-education paramedic degrees. It is important for this staff group to have a clear, holistic and deep understanding of what they are trying to achieve and why. There must be an expectation of an understanding of the wider sociological and cultural phenomena to facilitate the development of those learners, whether pre- or post-registration, within their locus. To achieve this, some thought must be given to the trajectory of the profession, the claimed scope and territory that the field of paramedicine purports to inhabit and the philosophical considerations of the consequences of actions or inactions in the discharge of their work. This appreciation, and indeed space for thinking, is not easy. And as can be seen in a wide range of examples, it must be done intentionally. As we have all no doubt experienced, it is all too easy for us to default to our well-worn historical areas of safety – the ambulance context.

## (Un)intended consequences: clinging to historical practices, divergence and the path of least resistance

As emergency driving theory advises, look for the path of least resistance to continue making progress. Before we know it, our educational programmes, designed with the best of intentions, all too easily frame examples in theoretical or practical teaching sessions and in assessments through an ‘ambulance’ or ‘pre-hospital’ (or should that more accurately be ‘out-of-hospital’?) lens, perhaps unintentionally using colloquial language that is readily understood and repeated by learners who have had exposure to ambulance-based, practice-based learning (PrBL), perhaps with conflicting messages between what is taught in theory and experienced in PrBL (Worsfold et al., 2024). Is this unintentional or intentional? Significantly, given the regulatory frameworks governing paramedicine, and more specifically, regulator-approved paramedic education programmes, which allow for divergence in approach, design and ethos of approved programmes, provided they meet the regulator’s relevant standards, there is a spectrum of design, approach, ethos and pedagogy between programmes. Despite this, however, one hallmark of pre-registration paramedic education in the UK is the high proportion of practice-based learning (PrBL) in such courses. Often, and in keeping with the pre-registration curriculum guidance (known as the ‘Paramedic Curriculum’) of the Royal College of Paramedics (RCoP), these courses are often based 50% on PrBL. However, divergence across the UK is evident in the make-up of this PrBL offering. While the RCoP Paramedic Curriculum and associated Programme Management Guidance (PMG) recommend baseline experiences, there is a historical precedence, in keeping with the roots of the profession, that has meant emergency ambulance service exposure is the majority, if not almost exclusive, domain for PrBL. In recent years, there has been some development of this area, with RCoP guidance now recommending baseline experiences in ‘non-ambulance’ areas. However, this is not compulsory and certainly not required by the regulator. This means that pre-registration paramedic PrBL is provider-specific and will often depend on local factors, commissioning arrangements, political will and both faculty and wider health sector positionality, rather than being a reflection exclusively of educationally underpinned theory. This provides divergence between those programmes that offer little or no ‘non-ambulance’ experience (perhaps a few days or a week, if any) and those who endeavour to match ‘ambulance sector’ experience like for like with ‘cross-sector’ experience, such as the BSc Paramedicine at Robert Gordon University, Aberdeen (Brogan & Green, 2022). (Here, I declare an interest as Principal Lecturer, Paramedicine at RGU.)

As with many aspects in our fledgling profession, we have not yet truly explored this phenomenon. With the expectations to consider these points (which are by no means the limit of expectation we place on those in an educational arena), while we have initially explored this in the context of higher education, the requirements outlined (and therefore, the expectations also) must surely branch wider than to what we might regard as the formal ‘teachers’ of paramedicine. Instead, this is a topic that must concern all of us within and associated with our profession. While we are just scratching the surface of these questions, and starting to consider more closely what it is to be a paramedic and what the trajectory of our profession will be (Brogan, 2026b), we must resolve to confront and explore these questions head on and engage with the State in their quest to answer these questions too (Brogan, 2026c; House of Lords Public Services Committee, 2026). For ourselves (each of us, at all levels) and our profession, to enable whatever the trajectory looks like that we choose based upon society’s needs of the profession, we do this purposively (Rodgers & Antony, 2023).

## We need to talk: confronting the elephant(s) in the room

There is no doubt that our profession faces many difficult questions, and every one of us must consider these points if we are to determine the profession’s future. But we must be open to differing views, the changing landscape that our profession faces and the changing demands and expectations of the public. Haidt (2006) introduces us to the metaphor of the elephant and the rider, the elephant being the emotional, instinctive ‘power’ that must be directed by the rational rider, who can see the path ahead. These phenomena can also be articulated through ‘thinking fast and slow’ (Kahneman, 2011), or the ‘chimp paradox’ (Peters, 2012), among others. One thing we must ensure is that our elephant doesn’t overpower our rider, merely due to entrenched historical and cultural views. We must all face head on the questions of determining the trajectory of the profession (Brogan, 2026b), the potential tyranny of historical, underlying, culturally fetishised and institutionalised legacy approaches (Corman et al., 2025) and acknowledgement of the phenomenon that is developing in pre-registration paramedic admissions – aspirant learners who have no intention or interest in practising in an ambulance sector environment but have a passion to practise as a paramedic in one of the many other domains where paramedics now operate effectively. Our ‘safe-space’ default to rooting educational experiences through an ‘ambulance’ lens can, if not acknowledged, transcend all elements of programmes, not merely in ‘formal’ educational experiences but in terms of structural and cultural expectations, including the uniform or dress code that learners might be expected to adopt (Brogan, 2026a). It is notable that in Scotland, all pre-registration paramedic students’ base uniform consists of a grey polo shirt and blue trousers – purposely chosen to reflect the Scottish national colour scheme for Allied Health Professional (AHP) students. Whereas this is a physical representation of ‘not all paramedics wear green’ (Eaton, 2019), surely this must have some impact on the framing and belonging of, and wider cultural impact on, learners, paramedics and the public. This is not to mention the stark visual difference from other pre-registration paramedic students from other parts of the UK.

## Riding the elephant: where do we start?

We must all face up to the continued ‘de-coupling’ of paramedicine from the ambulance sector work environment (Corman et al., 2025) and challenge our own practices, which, if we scratch the surface, are likely to reveal a tyranny of entrenched and subconscious currents.

Now that the new term is about to start, we (all of us, not merely those who ‘work in education’) must pledge to ourselves that we will make space to consider and discuss these considerations (and the many more not outlined in this piece), to purposefully set the trajectory for our profession, and ultimately to define our profession’s offering to society. Now is the time to take a good long, hard look at ourselves. Who are we? Where are we going? And are the ways of working that we cling to helping or hindering us? Let’s confront these elephants in the room by first ensuring our rider is in control.

## Conflict of interest

JB is Consultant Editor for the *Journal of Paramedic Practice*.

## References

[bibr_1] Brogan J. (2026a). *Re-defining the Paramedic’s offer to society: Leading change at Robert Gordon University (RGU)*. International Conference on Public Health and Preventive Medicine, Barcelona, Spain, Creovate Conferences.

[bibr_2] Brogan J. (2026b). Talking trajectories: Where are we aiming? *Journal of Paramedic Practice*, 18(6), 225. https://doi.org/10.12968/jpar.2026.0063.

[bibr_3] Brogan J. (2026c). *Written evidence to the House of Lords Public Services Committiee Inquiry on the role of ambulance services in supporting accident and emergency department capacity of James Brogan (Principal Lecturer, Paramedicine at Robert Gordon University) (AMB0006)*. House of Lords Public Services Select Committee. https://committees.parliament.uk/writtenevidence/165691/pdf/.

[bibr_4] Brogan J. & Green L. (2022). *Undergraduate paramedic placements in a remote island health board*. College of Paramedics National Conference 2022. https://rgu-repository.worktribe.com/output/2048942.

[bibr_5] Brooks I. A. Cooke M. Spencer C. et al (2016). A review of key national reports to describe the development of paramedic education in England (1966–2014). *Emergency Medicine Journal*, 33(12), 876. https://doi.org/10.1136/emermed-2015-205062. 26643926 10.1136/emermed-2015-205062

[bibr_6] Corman M. K. Phillips P. & McCann L. (2025). The future of paramedic education: Problematizing the translucent curriculum in paramedicine. *Paramedicine*, 22(4), 205–214. https://doi.org/10.1177/27536386251338525.

[bibr_7] Eaton G. (2019). Paramedic. *noun*. *British Paramedic Journal*, 4(2), 1. https://doi.org/10.29045/14784726.2019.09.4.2.1. 10.29045/14784726.2019.09.4.2.1 PMC7706760 33328830

[bibr_8] Haidt J. (2006). *The happiness hypothesis: Putting ancient wisdom and philosophy to the test of modern science*. Random House. https://doi.org/10.1007/s10902-007-9049-2.

[bibr_9] House of Lords Public Services Committee. (2026). *The role of ambulance services in supporting accident and emergency department capacity*. UK Parliament. https://committees.parliament.uk/publications/53610/documents/299350/default/.

[bibr_10] Kahneman D. (2011). *Thinking, fast and slow*. Allen Lane and Penguin Books.

[bibr_11] Millar E. (1966). *Report by the Working Party on Ambulance Training and Equipment. Part 1 – Training*. Ministry of Health, Scottish and Home Department. HMSO. https://wellcomecollection.org/works/sdbkcyec/items.

[bibr_12] Millar E. (1967). *Report by the Working Party on Ambulance Training and Equipment: Part 2 – Equipment and Vehicles*. Ministry of Health, Scottish and Home Department. HMSO.

[bibr_13] Peters S. (2012). *The chimp paradox*. Vermilion.

[bibr_14] Rodgers B. & Antony J. (2023). In pursuit of a culture of continuous improvement: Scotland’s National Ambulance Service. *International Journal of Public Administration*, 46(9), 636–646. https://doi.org/10.1080/01900692.2021.2009857.

[bibr_15] Worsfold M. E. Jouanny C. Hamer E. et al (2024). ‘This is how I’m going to do it, but this is not how you’re going to do it’: The expectation gap between student paramedics and mentors in East and Central Scotland. *BMC Medical Education*, 24(1), 368. https://doi.org/10.1186/s12909-024-05319-z. 38570785 10.1186/s12909-024-05319-z PMC10993586

